# Cell-Penetrating Peptides Enhance the Activity of Human Fibroblast Growth Factor 2 by Prolonging the Retention Time: A New Vision for Drug-Delivery Systems

**DOI:** 10.3390/ijms21020442

**Published:** 2020-01-10

**Authors:** Jaehoon Lee, Mijin Kwon, Naeun Oh, Jaehyun Park, Sangkyu Park, Jeongmin Seo, Sangho Roh

**Affiliations:** 1 Biomedical Research Institute, NeoRegen Biotech Co., Ltd., Gyeonggi-do 16614, Korea; jaylee6322@gmail.com (J.L.); good0039@hanmail.net (S.P.); 2 Cellular Reprogramming and Embryo Biotechnology Laboratory, Dental Research Institute, Seoul National University School of Dentistry, Seoul 08826, Korea; rnjsalwls23@hanmail.net (M.K.); onajun@naver.com (N.O.); 680087@hanmail.net (J.P.)

**Keywords:** cell-penetrating peptides, drug-delivery system, Ara27, re-release, fibroblast growth factor 2

## Abstract

Cell-penetrating peptides (CPPs) are defined by their ability to deliver cargo into cells and have been studied and developed as a promising drug-delivery system (DDS). However, the issue of whether the CPPs that have already entered the cells can be re-released or reused has not been studied. The purpose of this research was to construct CPP-conjugated human fibroblast growth factor 2 (hFGF2) and investigate whether they can be re-released from the cell membrane for reuse. This study combined hFGF2 with Tat or Ara27, a newly developed CPP derived from the zinc knuckle (CCHC-type) family protein of *Arabidopsis*. Human dermal fibroblast (HDF) was treated with Tat-conjugated hFGF2 (tFGF2) and Ara27-conjugated hFGF2 (NR-FGF2) for both long and short durations, and the effects on cell growth were compared. Furthermore, tFGF2 and NR-FGF2 re-released from the cells were quantified and the effects were evaluated by culturing HDF in a conditioned medium. Interestingly, the proliferation of HDF increased only when NR-FGF2 was treated for 1 h in endocytosis-independent manner. After 1 h, NR-FGF2 was significantly re-released, reaching a maximum concentration at 5 h. Furthermore, increased proliferation of HDF cultured in the conditioned medium containing re-released NR-FGF2 was discovered. While previous studies have focused on the delivery of cargo and its associated applications, this study has revealed that combinations of superior CPPs and therapeutics can be expected to prolong both the retention time and the cell-penetrating capacity, even in the presence of external factors. Therefore, CPPs can be applied in the context of topical drugs and cosmetics as a new DDS approach.

## 1. Introduction

Although advances in drug screening research have enabled the identification of candidates with desired therapeutic effects, delivering a drug to a specific target to maximize its effect has proven to be particularly challenging. For example, although the effects of the candidates are expected to be potentially superior at the action site, their ability can be limited because of the drug’s physicochemical properties (e.g., shape, size, charge, and hydrophobicity or unwanted enzymatic processes) [1]. Drug-delivery systems (DDSs) have been extensively studied as potential tools for improving the therapeutic efficacy by significantly enhancing the pharmacokinetic and pharmacodynamic properties and reducing undesired effects [2]. DDSs are used to deliver therapeutics for the following purposes: (i) to change a drug’s properties and improve its solubility [3], (ii) to reduce toxicity and immunogenicity [4], (iii) to prolong the drug’s circulation time in the body [5], (iv) to achieve specific targeting [6], and (v) to adjust drug release [7]. Therefore, various creative attempts using DDSs are expected to contribute to the advancement of the therapeutic field.

In order to maximize the efficiency of the drug, various DDS vehicles (e.g., liposomes, hydrogels, nanoparticles, and micelles) have been developed. One such channel is cell-penetrating peptides (CPPs), defined by their ability to cross cell membranes without specific receptors and to carry cargo into cells. CPPs have been studied and developed as a promising DDS strategy since the first discovery of the HIV-1 transactivating protein known as Tat [8,9]. These CPPs have been used to transfer nucleic acids [10], proteins [11], small-molecule therapeutics [12], fusion complexes [13], and quantum dots [14]. Various applications have been attempted based on the cell permeability of these peptides. When low-molecular-weight protamine (LMWP) was combined with human fibroblast growth factor 2 (hFGF2) or human vascular endothelial growth factor, cell permeability was significantly increased, suggesting that it could be used as an effective percutaneous delivery system [15]. The anticancer effect in vivo was confirmed using a fusion complex composed of triplicated Tat, siRNA, and target-specific aptamers [16]. In addition, the efficiency of CRISPR was increased by delivering Cas9 complexed with guide RNA intracellularly using a Tat adaptor system [17]. Preclinical evaluations by CPP-applied therapeutics in a variety of disease models have indicated promising results, thus providing basic evidence for clinical trials [18,19,20]. 

However, there are still several obstacles such as lack of specificity for the target, immunogenicity, cytotoxicity, and instability [21]. In addition, most CPPs require high treatment concentrations for cell permeation even in an in vitro environment [22]. The complicated mechanisms associated with CPPs also indicate that they may not work as expected under certain conditions. It has been revealed in various studies that the cell-entry mechanisms of CPPs depend on specific types of peptides and environments [23]. For example, nona-arginine (Arg_9_) enters the cells via energy-dependent endocytosis at low concentrations; at high concentrations, it penetrates the cell membrane directly in an energy-independent manner [24,25]. At concentrations of 2 μM, Arg_9_ can enter the cells via endocytosis at 37 °C, but not at room temperature. Additionally, it is unclear whether the CPP-conjugated cargo that has already permeated the cells can be re-released from the cells. Therefore, it is necessary to develop CPPs that are more resistant to environmental effects (even at low concentrations) and to understand the positioning of the CPPs after cell penetration. 

In a previous study, the development of Ara27 CPPs has been examined, which are derived from a zinc knuckle (CCHC-type) family protein of *Arabidopsis* and function at low concentrations with short treatment times. Table 1 presents comparisons between the physicochemical properties of Ara27 and Tat. The objective of this paper was to construct and assess the effects of Ara27-conjugated hFGF2 (NR-FGF2) and determine whether they can be re-released from the cell’s membrane and reused. CPP-conjugated hFGF2 was purified by his-tag affinity chromatography, and the activity and cell-penetrating ability were analyzed. Human dermal fibroblast (HDF) was treated with control hFGF2 (cFGF2), Tat-conjugated hFGF2 (tFGF2), and NR-FGF2 for a short time to investigate the short-term treatment effects. The results showed that only NR-FGF2 significantly increased the cell viability of HDF, and the interaction between CPPs and the cell membranes did not contribute to the results by using heparin which was known to block the effects of CPPs. Moreover, as a result of further experiments using endocytosis inhibitors, it was confirmed that the short-term treatment effects of NR-FGF2 was not related to the endocytosis pathway. The proliferation of HDF cultured in the conditioned medium containing re-released NR-FGF2 was increased, suggesting that NR-FGF2 could re-released into the medium and be reused. 

## 2. Results

### 2.1. Purification of CPP-Conjugated hFGF2

**Figure 1:**
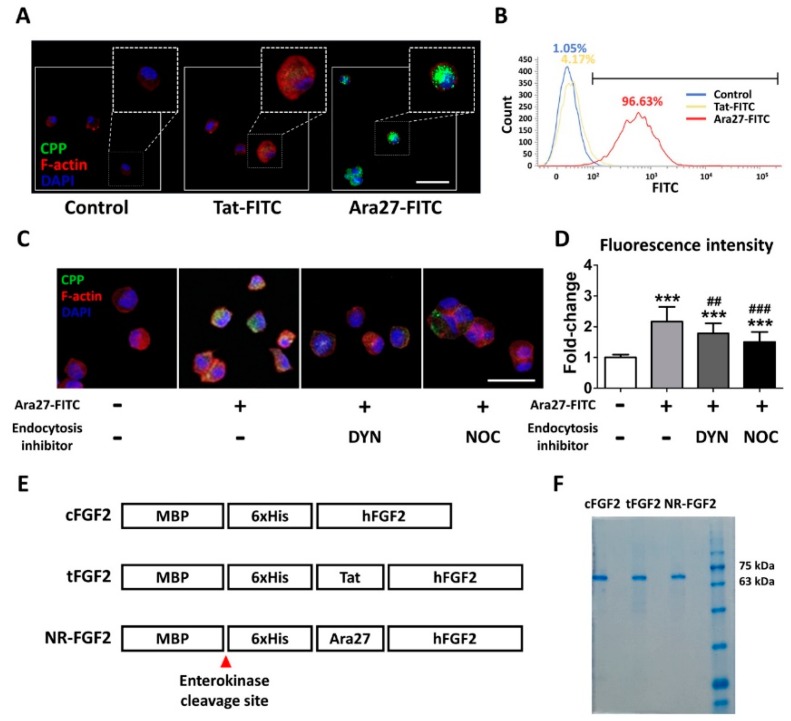
A newly developed cell-penetrating peptide (CPP), Ara27, and the purification of CPP-conjugated human fibroblast growth factor 2 (hFGF2). (**A**) Human dermal fibroblast (HDF) was treated with Tat-fluorescein isothiocyanate (FITC) and Ara27-FITC at 1 μM for 1 h; consequently, more Ara27-FITC than Tat-FITC enters the cells (scale bar = 50 μm, white). (**B**) The cell-penetrating ability of Ara27-FITC was also validated by fluorescence activated cell sorter. (**C**,**D**) The mechanism of the cell-penetrating ability of Ara27 was confirmed by dynasore and nocodazole (*n* > 80) (**E**) Control hFGF2 (cFGF2), Tat-conjugated hFGF2 (tFGF2), and Ara27-conjugated hFGF2 (NR-FGF2) were composed of maltose binding protein (MBP), his-tag for purification, CPPs, and functional hFGF2. (**F**) Fusion proteins were purified via Ni-NTA affinity chromatography and were analyzed by sodium dodecyl sulfate-polyacrylamide gel electrophoresis (SDS-PAGE). The results are the means of at least three independent experiments (mean + SD). *** p<0.001 versus the control group and ## p<0.01 and ### p<0.001 versus the Ara27-FITC-treated group.

In order to compare the permeability, HDF was treated at 1 μM for 1 h with Ara27-fluorescein isothiocyanate (FITC) and Tat-FITC before being analyzed by immunofluorescence staining and fluorescence activated cell sorting (FACS). The results indicated that Ara27-FITC translocated into the cells more than Tat-FITC (Figure 1A,B). To determine whether the cell permeability of Ara27 was caused by endocytosis, Ara27-FITC was treated in HDF after pretreatment with dynasore (DYN) and nocodazole (NOC), known as endocytosis inhibitors. As a result, despite treatment with endocytosis inhibitors, cell-penetrating effects still remained (Figure 1C). However, the effects were reduced as compared with only Ara27-FITC without inhibitors (Figure 1D). To confirm that Ara27 could also deliver macromolecular cargo, *Escherichia coli* (*E. coli*) BL21 were transformed to produce recombinant proteins (as shown in Figure 1E). After purification with his-tag affinity chromatography, the purified fusion proteins were confirmed by sodium dodecyl sulfate-polyacrylamide gel electrophoresis (SDS-PAGE) and Coomassie Blue staining (Figure 1F). 

### 2.2. Effects of CPP-Conjugated hFGF2 on HDF Cell Growth

**Figure 2:**
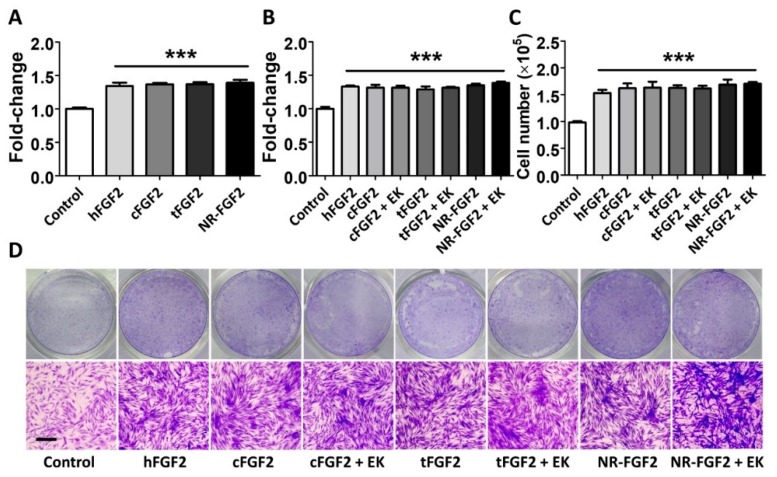
Effects of CPPs or MBP of fusion proteins on hFGF2 activity during long-term culture. HDF was cultured with normal hFGF2 and CPP-conjugated hFGF2 for five days and analyzed using a WST-1 cell viability assay and Crystal Violet staining. (**A**) tFGF2 and NR-FGF2 did not show significant differences to hFGF2. (**B**) The presence of MBP did not affect the hFGF2 activity. (**C**,**D**) This effect was also confirmed by counting the cell number and Crystal Violet staining (scale bar = 100 μm, black). The results are the means of at least three independent experiments (mean + SD). *** p<0.001 versus the control group.

In order to confirm the effect of purified CPP-conjugated hFGF2 on HDF cell growth, normal hFGF2, cFGF2, tFGF2, and NR-FGF2 were treated for five days in HDF at 1 nM before cell viability was analyzed. As a result, despite the combination of CPP and hFGF2, there was no significant difference between hFGF2 and tFGF2/NR-FGF2 (Figure 2A). In order to examine how the maltose binding protein (MBP) site of fusion proteins affects the hFGF2 activity, HDF was treated with the fusion proteins from which the MBP site was removed by EK. The cell viability and the number of the cells were significantly increased regardless of the presence of the MBP site (Figure 2B,C). Crystal Violet staining was used to confirm that the conjugation of CPP and the presence of MBP were not related to the hFGF2 activity (Figure 2D).

### 2.3. Effects of Short-Term Treatment of NR-FGF2 on HDF

**Figure 3:**
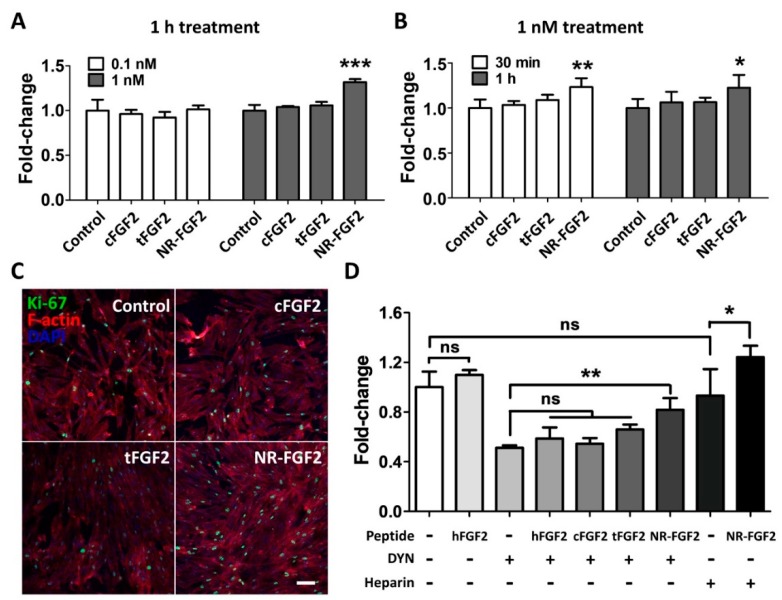
Effects of short-term treatment of NR-FGF2 on HDF. HDF was treated with cFGF2, tFGF2, and NR-FGF2 at 0.1 nM or 1 nM for 1 h and maintained for five days. (**A**) The proliferation of HDF significantly increased only when NR-FGF2 was treated at a concentration of 1 nM. (**B**) When CPP-conjugated hFGF2 was treated for 30 min, cell proliferation significantly increased only in HDF pretreated with NR-FGF2. (**C**) The expression of Ki-67 in the nucleus increased when NR-FGF2 was treated for a short time (scale bar = 100 μm, white). (**D**) Despite inhibiting the endocytosis pathway, the short-term treatment ability of NR-FGF2 was maintained. The results are the means of at least three independent experiments (mean + SD). * p<0.05, ** p<0.01, and *** p<0.001 versus the control group. ns, not significant.

In order to determine the effect of short-term CPP-conjugated hFGF2 treatment, HDF was treated with cFGF2, tFGF2, and NR-FGF2 at 0.1 nM or 1 nM for 1 h and maintained for five days. Interestingly, the WST-1 cell viability assay revealed that the HDF viability was significantly increased only when NR-FGF2 was treated at a concentration of 1 nM (Figure 3A). Moreover, when CPP-conjugated hFGF2 was treated for 30 min, cell proliferation was significantly increased only in HDF treated with NR-FGF2 (Figure 3B). In addition, it was confirmed that, as a result of labeling the proliferation marker Ki-67 by immunofluorescence staining, the expression of Ki-67 in the nucleus increased when NR-FGF2 was treated for a short duration (Figure 3C). To investigate whether the short-term treatment effect of NR-FGF2 was caused by endocytosis or strong interactions between CPPs and cell membranes, HDF was pretreated with DYN or washed with PBS containing heparin. The results showed that, in the case of washing with heparin, the cell viability was increased when NR-FGF2 was treated for a short time (Figure 3D). In addition, despite suppressing endocytosis of cells, short-term treatment of NR-FGF2 significantly increased proliferation of HDF. These results suggest that NR-FGF2 could enhance the proliferation of HDF despite receiving only short-term treatment regardless of the association between CPPs and membrane, or the endocytosis process. 

### 2.4. Cell-Penetrating Ability of NR-FGF2

**Figure 4:**
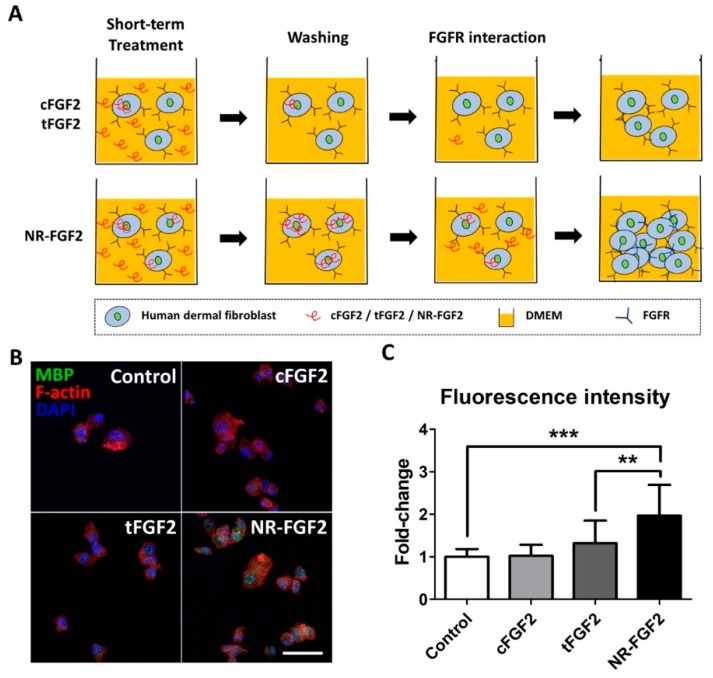
Cell-penetrating ability of NR-FGF2. (**A**) The enhanced cell-penetrating ability of Ara27 increased the residence time of hFGF2 and, second, that this could continue to affect cells (**B**,**C**) As a result of the short-term treatment of CPP-conjugated hFGF2, NR-FGF2 sufficiently penetrated the cells within 1 h (*n* > 80; scale bar = 500 μm, white). The results are the means of at least three independent experiments (mean + SD). ** p<0.01 and *** p<0.001 versus the control group.

In order to explain the effects of the short-term treatment of NR-FGF2, it was hypothesized in this study, first, that the enhanced cell-penetrating ability of Ara27 increased the residence time of hFGF2 and, second, that this could continue to affect cells (Figure 4A). Therefore, after washing, hFGF2 (which had already penetrated the cells) would be released out of the medium and would interact with HDF. For confirmation, HDF was treated with CPP-conjugated hFGF2 at 1 nM for 1 h; the cell permeability of each fusion protein was then confirmed by immunofluorescence staining. MBP was used as a specific marker to detect CPP-conjugated hFGF2, since cFGF2, tFGF2, and NR-FGF2 had MBP domain. The results revealed that, during short-term treatment, more cell permeation was evident with NR-FGF2 than with cFGF2 and tFGF2 (Figure 4B,C).

### 2.5. Re-Release of NR-FGF2 which Penetrated HDF

**Figure 5:**
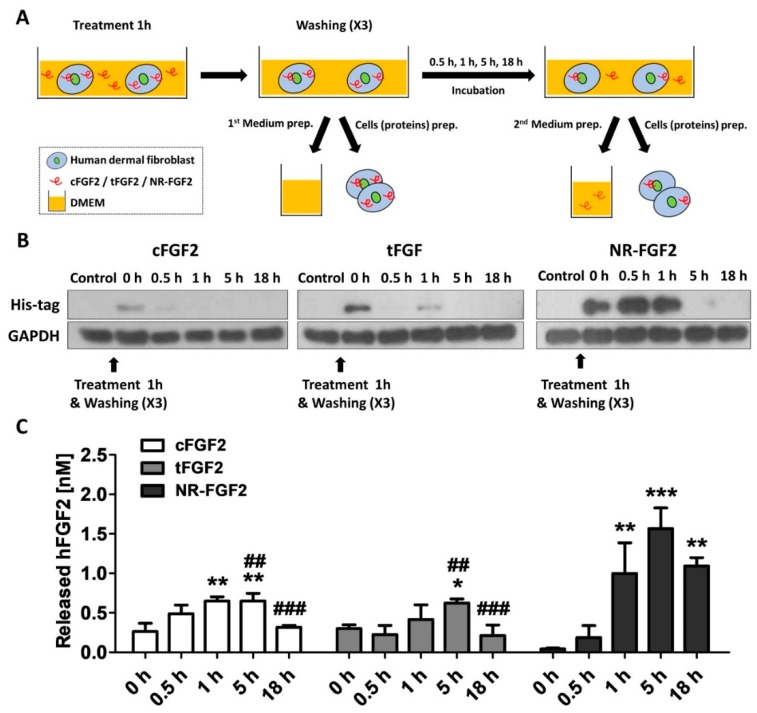
Re-release of NR-FGF2 that had penetrated HDF. (**A**) Experimental schematic diagram: (i) CPP-conjugated hFGF2 was treated in HDF for 1 h; (ii) after washing, the medium and cell lysate were harvested for presenting the initial (0 h) amount of hFGF2; (iii) the medium and cell lysate were obtained by culture time (0.5, 1, 5, and 18 h); and (iv) hFGF2 in the medium or cell lysate was analyzed by ELISA or western blot method, respectively. (**B**) NR-FGF2 remained in the cells for up to 5 h, whereas cFGF2 and tFGF2 were absent after 1 h. (**C**) At 1 h, NR-FGF2 was significantly re-released as compared with cFGF2 and tFGF2, and the concentration was maximized at 5 h. The results are the means of at least three independent experiments (mean + SD). * p<0.05, ** p<0.01, and *** p<0.001 versus the control group and ## p<0.01 and ### p<0.001 versus the NR-FGF2-treated group.

In order to investigate the release of cell-permeated NR-FGF2, HDF was treated with CPP-conjugated hFGF2 for 1 h, and the medium and cell lysate were obtained by culture time (Figure 5A). To confirm how the cell-penetrated hFGF2 was maintained over time, hFGF2 of the cell lysate was analyzed using the Western blot method. It was also confirmed that more cell penetration initially occurred with NR-FGF2 than with cFGF2 and tFGF2 (as revealed by the data in Figure 4B), and enough remained present in the cells for 1 h. Surprisingly, the presence of NR-FGF2 was detected in the cells for up to 5 h, whereas cFGF2 and tFGF2 were absent after 1 h (Figure 5B). To confirm the release of cell-penetrated NR-FGF2 into the medium, the amount of hFGF2 in the medium was quantified by enzyme-linked immunosorbent assay (ELISA) analysis. It was revealed that all fusion proteins were re-released into the medium after 1 h (Figure 5C). Significant re-release of NR-FGF2 was evident after 1 h (as compared with cFGF2 and tFGF2) and the maximum concentration was achieved at 5 h. Furthermore, the re-released NR-FGF2 was maintained at a concentration of 1 nM or more for a duration of 18 h. 

**Figure 6:**
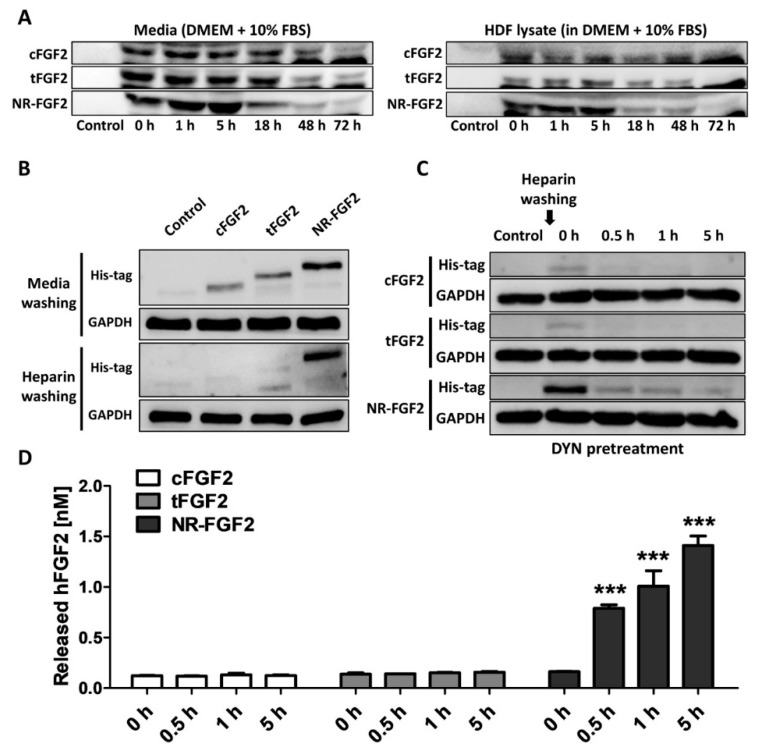
Mechanism of short-term treatment effects of NR-FGF2. (**A**) CPP-conjugated hFGF2 existed for at least 72 h under experimental conditions. (**B**) It was confirmed that the short-term treatment ability of NR-FGF2 was not due to its binding to the cell membrane by heparin washing. (**C**) The inhibition of endocytosis pathways did not affect cell penetration of NR-FGF2. (**D**) Re-released NR-FGF2 was increased over time and the maximum concentration was achieved at 5 h. The results are the means of at least three independent experiments (mean + SD). *** p<0.001 versus the control group.

To confirm whether the results were caused by re-release of NR-FGF2 or simple degradation under experimental conditions, CPP-conjugated hFGF2 was added to the medium or cell lysate, and its stability was analyzed. As a result, it was confirmed that although the amounts were gradually decreased in the medium and the cell lysate-containing medium, CPP-conjugated hFGF2 existed by 72 h (Figure 6A). In addition, in order to exclude the effects of NR-FGF2 that could not be washed by binding to the cell outer membranes, HDF was treated with CPP-conjugated hFGF2 for 1 h at 100 nM and washed with PBS containing heparin three times. As a result of Western blot analysis, it was confirmed that NR-FGF2 translocated into cells more than cFGF2 and tFGF2 (Figure 6B). To confirm that the interaction between CPPs and membranes, and endocytosis pathways do not contribute to the short-term treatment effects of NR-FGF2, the release of NR-FGF2 was analyzed using heparin and DYN. The results showed that NR-FGF2 penetrated the cells in a short time and the amount was decreased over time, indicated that NR-FGF2 entered the cells regardless of the endocytosis pathway (Figure 6C). Correspondingly, re-released NR-FGF2 was increased over time and the maximum concentration was achieved at 5 h (Figure 6D). Thus, the interaction between CPPs and cell membrane, and endocytosis pathway were not major factors of short-term treatment effect of NR-FGF2. 

### 2.6. Effects of Re-Released NR-FGF2

**Figure 7:**
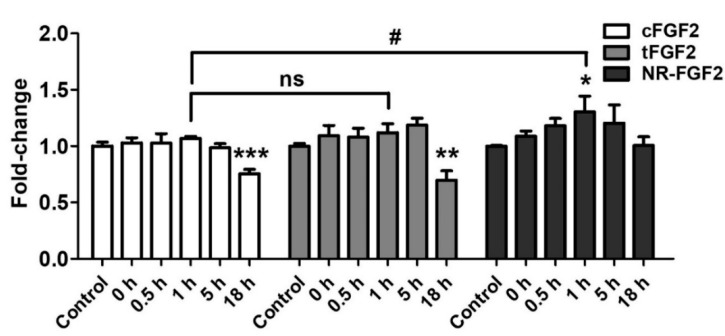
Effects of re-released NR-FGF2. CPP-conjugated hFGF2 was pretreated at 1 nM for 1 h, and HDF was cultured in the conditioned medium obtained at a specific time for five days. The results showed that only the cell growth of HDF cultured in the conditioned medium obtained 1 h after the pretreatment of NR-FGF2 was significantly increased. The results are the means of at least three independent experiments (mean + SD). * p<0.05, ** p<0.01, and *** p<0.001 versus the control group and # p<0.05 versus the NR-FGF2-treated group. ns, not significant.

In order to investigate the influence of re-released NR-FGF2 on increasing HDF growth, CPP-conjugated hFGF2 was pretreated in HDF for 1 h, at 1 nM, and the cells was cultured for five days as described above. During the culture period, the conditioned medium in which CPP-conjugated hFGF2 was expected to be re-released was obtained at specific time points. Newly seeded HDF was cultured in the conditioned medium for five days and analyzed by WST-1 cell viability assay. As a result, only the HDF that was cultured in the conditioned medium (obtained 1 h after the pretreatment of NR-FGF2) significantly increased cell growth (Figure 7). Conversely, the proliferation of the HDF that was cultured in the conditioned medium obtained 18 h after the pretreatment of cFGF2 and tFGF2 was significantly reduced. There was no significant difference between the control group and the group that was cultured in the NR-FGF2 18 h conditioned medium. Therefore, these results confirm that re-released NR-FGF2 can increase the proliferation of HDF.

## 3. Discussion

The current interest in the field of DDS is focused on the effective delivery of a drug to its desired target [26]. Advances in DDS enable a drug to reach its target tissue, but insufficient uptake in the diseased target cell can result in limited therapeutic effects [1]. Consequently, CPPs have been recognized as a suitable tool for DDS because of their ability to deliver cargo intracellularly without eliciting an immune response [27]. Although various CPPs have been developed for application in animal models and clinical trials, research is still ongoing to find more effective CPPs [18]. Studies have shown that widely used CPPs such as Penetratin, Arg_8_, Tat, and Transportan require high concentrations (>5 μM) for cell permeation [28,29]. In a previous study, it was confirmed that Ara27 demonstrated higher cell permeability as compared with Tat and membrane-translocating motif [30,31] at 100 to 200 nM. Consistent with previous study, Ara27-FITC had extremely higher cell-penetrating ability than Tat-FITC at 1 μM (Figure 1C,D). In order to verify the cargo delivery capacity of Ara27, MBP-Ara27-conjugated hFGF2 was constructed and purified (Figure 1E,F). The MBP that was used to enhance the solubility and production was maintained during subsequent experiments to evaluate the cargo delivery capacity of Ara27. It was revealed that MBP did not affect the activity of hFGF2, despite the greater molecular weight of MBP (2.5 times greater than hFGF2) (Figure 2B–D). Furthermore, Ara27 was shown to transport macromolecules comprising hFGF2 and MBP into cells.

Scarring is one of the most common but undesirable complications during wound healing, which may lead to potentially severe disfigurement, functional impairment, and mental trauma; however, there is no definitive treatment available so far [32]. Because skin-wound healing is a complex interaction process involving cytokines, growth factors, and extracellular components, appropriate treatment methods for scar-free skin regeneration are required, which must be based on an accurate understanding of the mechanisms involved [33]. Recent studies have shown that hFGF2 promoted skin regeneration at wound sites on rabbits’ ears and decreased scar formation by upregulating the Notch1/Jagged1 signaling pathway, thereby inhibiting the differentiation of epidermal stem cells to myofibroblasts [34]. As hyperplasia of myofibroblasts is the main cause of scarring, hFGF2 can be a potential therapeutic to inhibit scar formation [35]. However, the skin’s barrier and the epidermis can prevent hFGF2 from accessing the fibroblast growth factor receptor (FGFR) on epidermal stem cells or on skin fibroblasts in the dermis [36]. Therefore, studies were conducted to increase the skin’s permeability by combining recombinant epidermal growth factor (EGF) and hFGF2 with LMWP, which is a nontoxic arginine-rich CPP [15,37]. Consistent with LMWP-EGF experiments, CPP-conjugated hFGF2 had no significant effects on HDF as compared with normal hFGF2. It was reported that hFGF2 increased the proliferation of HDF by binding to FGFR on cell membranes and subsequently activating the ERK1/2 and JNK pathways [38]. Therefore, the conjugation of CPP with ligands that interact with the receptors on the cell membrane can have little or no effect.

Surprisingly, when screening for the treatment time and concentration of NR-FGF2, we found in this study that only NR-FGF2 could significantly increase the proliferation of HDF during short-term treatment (Figure 3A,B). Correspondingly, the expression level of Ki-67 (known as the proliferation marker) was also increased only during short-term treatment with NR-FGF2 (Figure 3C). In order to explain the results, it was hypothesized that effective CPPs can help both maintain the long-term effects of cargo on cells and translocate cargo into the cells, even if external factors are present (e.g., washing, contact, and dilution). It was expected that hFGF2 that was permeated into the cells by Ara27 within a short timeframe would be re-released and reused after washing (Figure 4A). First, it was confirmed that NR-FGF2 sufficiently penetrated the cells within 1 h (Figure 4B,C). Studies have shown that peptide degradation can commence immediately after uptake and can be mostly completed in 1 to 2 h [39,40]. Consistent with previous studies, it was revealed that most of the tFGF2 and NR-FGF2 were largely degraded within 2 h, whereas NR-FGF2 appeared to be present in small amounts even at 5 h (Figure 5B). This supports previous data (Figure 4B) showing that, within 1 h, more cell penetration by NR-FGF2 was evident than with tFGF2. It was also confirmed that the amount of NR-FGF2 in the medium increased in accordance with the amount of NR-FGF2 that decreases in the cells with time (Figure 5C). The results showed that CPP-conjugated hFGF2 was maintained for more than 48 h in the medium containing cell lysate, indicating that NR-FGF2 was not just simply degraded (Figure 6A). Heparin and dextran sulphate have been reported to completely inhibit the ability of CPPs by spontaneously binding with CPPs [41]. Moreover, heparin is also known to specifically bind to hFGF2, increasing the stability and efficacy of hFGF2 [42]. Therefore, heparin is a proper tool to prevent further cell permeation of CPPs or to remove CPPs that have not yet penetrated into cells at certain time points. Further study using heparin showed that cFGF2 and tFGF2 were washed out through heparin, while NR-FGF2 was maintained (Figure 6B). These results indicated that the strong association between CPPs and cell membranes was not related to the short-term treatment effects of NR-FGF2 (Figure 3D). 

The uptake mechanisms of CPPs are divided into energy-independent uptake and energy-dependent uptake; interestingly, they are nonexclusive [23]. Although Ara27 maintained cell-penetrating ability, it was reduced by caveolae- and clathrin-dependent endocytosis inhibitors (Figure 1C,D). In preliminary studies using inhibitor, Ara27 penetrate the cell regardless of macropinocytosis or phagocytosis (data not shown). Thus, Ara27 enters the cell through caveolae- and clathrin-dependent endocytosis and by direct translocation. While studies on the macropinocytosis [43], endocytosis [44], and escape of the endosomes [45] of CPPs have been well established, various methods of passive uptake have been suggested which include: (i) transient pore [46], (ii) inverted micelle [47], (iii) domain and membrane defaults [48], and (iv) adaptative translocation [49]. However, the re-release or reuse of the CPPs that have entered the cells has not been fully explained. This study confirmed that NR-FGF2 rapidly penetrated the cells, and the amount decreased after retention for 1 h (Figure 5B). Correspondingly, it was established that, after 1 h, the concentration of the re-released NR-FGF2 in the medium increased above 1 nM; the proliferation of HDF cultured in this conditioned medium also significantly increased (Figure 5C and Figure 7), suggesting that cell-penetrated NR-FGF2 could be re-released and reused. The presence of NR-FGF2 in the medium can explain why the viability of HDF cultured in the 18 h conditioned medium of the NR-FGF2-treated group was the same as that of the control group, whereas the viability of HDF cultured in 18 h conditioned media from the cFGF2- or tFGF2-treated groups was reduced. Endocytosis inhibitors did not impair the short-term treatment effects of NR-FGF2 (Figure 3D and Figure 6C,D), which only indicated that NR-FGF2 did not undergo the series of processes (e.g., penetration and re-release) through the endocytosis pathway. In other words, although it seems clear that NR-FGF2 is permeated into the cells primarily in ATP-independent manner, it still remains unclear what conditions and mechanisms are involved in the release of CPPs. Therefore, further study is required to determine the accurate mechanisms of CPPs re-release. 

In conclusion, while previous research has focused on cargo delivery and its mechanisms and applications, this study has confirmed that combinations of superior CPPs and therapeutics can be expected to extend the duration of drug action and cell-penetrating capability, which suggests a new approach for applying CPPs in the context of DDS. In particular, it can be suitable for applications where the effects of drugs are hardly expected, such as skin barriers and blood-brain barrier, in that CPPs can help increase the cell penetration and the drug retention time at target sites. Although an understanding of the mechanisms of the re-release and reuse of CPPs and studies in the in vivo environment are still required, this research indicates that CPPs can be used in the context of topically applied drugs and cosmetics.

## 4. Materials and Methods 

### 4.1. General Materials

Ni-NTA resin and columns for purification and enterokinase enzyme were purchased from Takara Bio (Kusatsu, Japan) and GenScript (Piscataway, NJ, USA), respectively. DYN, NOC, and Alexa Fluor 647-conjugated phalloidin were acquired from Cayman Chemical (Ann Arbor, MI, USA). Antibodies were obtained from the following companies: Ki-67 and his-tag antibodies from Novus Biologicals (Centennial, CO, USA), maltose binding protein antibodies from Biorbyt (Cambridge, UK), IgG isotype control antibodies from Bioss (Woburn, MA, USA), and GAPDH antibodies from Santa Cruz Biotechnology (Dallas, TX, USA).

### 4.2. Peptide Synthesis

Tat and Ara27 were synthesized by LifeTein LLC (Hillsborough, NJ, USA). Subsequently, Tat and Ara27 were labeled with FITC; Tat-FITC and Ara27-FITC were dissolved in distilled water to prepare a 1 M stock solution. The sequence information details are documented in Table 2.

### 4.3. Analysis of the Physicochemical Properties of Tat and Ara27

Tat and Ara27 with uncharged N-termini (–NH_2_) and amidated C-termini (–CONH_2_) were used for all analyses of physicochemical properties. Calculations were made using PepCalc.com’s peptide property calculator (http://pepcalc.com; Innovagen AB, Lund, Sweden) and the Membrane Protein Explorer tool, version 3.3.0 (http://blanco.biomol.uci.e.,du/mpex/; Stephen White Laboratory) [50,51].

### 4.4. Cell Culture

HDF, which was obtained from the American Type Culture Collection (Manassas, VA, USA), was cultured in Dulbecco’s modified Eagle’s medium (DMEM) from GE (Boston, MA, USA), which contained 4.5 g/L D-glucose, 10% fetal bovine serum, and 1% penicillin/streptomycin. HDF was then cultured at 37 °C in an incubator containing a humidified atmosphere of 5% CO_2_. In order to determine the effect on the growth of the CPP-conjugated hFGF2, HDF was seeded in DMEM at a density of 1.0 × 10^4^ cells per well in 12-well plates. After 24 h, the medium was replaced with the CPP-conjugated hFGF2 at a concentration of 1 nM. After five days of culturing, the cells were stained with Crystal Violet and observed using an EVOS CL Core microscope (Life Technologies, Carlsbad, CA, USA) at 40× or 100× magnification.

### 4.5. Analysis of the Cell Permeability of CPPs

HDF was cultured and treated with Tat-FITC and Ara27-FITC at 1 μM for 1 h to assess the cell permeability. The cells were washed with heparin-containing phosphate-buffered saline (PBS) (which inhibited the interaction between the CPPs and the cell’s surface) and were detached with trypsin/ethylenediaminetetraacetic acid (EDTA). The cells were then fixed with 4% paraformaldehyde for 15 min. After washing with PBS, the cells were Alexa Fluor 647-conjugated phalloidin for 30 min. Following thorough washing, the cells were mounted on slides using a mounting solution with 4′,6-diamidino-2-phenylindole (DAPI, Thermo Fisher Scientific, Waltham, MA, USA) fluorescent stain. All slides were analyzed using confocal microscopy (LSM 800; Zeiss, Baden-Württemberg, Germany).

To analyze cell permeation mechanism of Ara27, HDF was cultured and pretreated with 50 μM DYN or 3 μM NOC for 24 h. After thoroughly washing, HDF was treated with Ara27-FITC at 1 μM for 1 h and the cells were analyzed using confocal microscopy as described above. Fluorescence intensity was analyzed by ZEN 3.1 (blue edition) provided by Zeiss.

### 4.6. FACS

HDF was cultured and treated with Tat-FITC and Ara27-FITC at 1 μM for 1 h. The cells were washed with heparin-containing PBS and were detached with trypsin/EDTA. The cells were then fixed with 4% paraformaldehyde for 15 min. Following thorough washing, the cells were analyzed by fluorescence activated cell sorter (FACSVerse; BD Biosciences, Franklin Lakes, NJ, USA).

### 4.7. Fusion Protein Design

The CPP-conjugated hFGF2 fusion protein comprises four parts which include: MBP, his-tag for purification, CPP, and the functional hFGF2. Tat was used as a positive control for the comparison of cell-penetrating ability. Table 2 documents the sequence information details. The molecular weights of cFGF2, tFGF2, and NR-FGF2 were 62.0, 64.5, and 66.4 kDa, respectively.

### 4.8. Purification of Fusion Protein

For the expression of the fusion protein, pMAL-c5E vectors containing genes encoding the target protein were constructed via GenScript. The designed vectors were transformed into *E. coli* BL21 competent cells for protein purification. The bacteria were aerobically cultured with 0.4 mM isopropyl β-D-1-thiogalactopyranoside at 200 rpm for 4 h in lysogeny broth (Hardy Diagnostics, Santa Maria, CA, USA). The harvested *E. coli* was disrupted by sonication, and the fusion protein was purified from the lysate using Ni-NTA affinity chromatography. Imidazole was removed by dialysis and endotoxin was removed by Acrodisc^®^ Units with Mustang^®^ E Membrane (Pall, Port Washington, NY, USA). The purified fusion proteins were analyzed by SDS-PAGE and Coomassie Blue staining. The proteins were then treated with enterokinase at a concentration of 1 U/μL and incubated overnight at 12 °C (according to the manufacturer’s instructions) in order to remove the MBP site from the fusion proteins.

### 4.9. Cell Viability Assay

HDF was seeded at a density of 1.0 × 10^3^ cells per well in 96-well plates. After 24 h, the medium was replaced with the CPP-conjugated hFGF2 at a concentration of 1 nM and maintained for five days. A WST-1 cell viability assay kit (Dongin LS, Seoul, Republic of Korea) was used to test the cell viability effects of the CPP-conjugated hFGF2. General hFGF2 was used as a positive control.

For further analysis, HDF was seeded at a density of 5.0 × 10^3^ cells per well in 24-well plates. After 24 h, the medium was replaced with the CPP-conjugated hFGF2 at a concentration of 1 nM and maintained for five days. The cells were analyzed using Crystal Violet staining or cell counting assay using hemocytometer.

### 4.10. Short-Term Treatment of CPP-Conjugated hFGF2

HDF was seeded in 12-well plates and 96-well plates (as described above) and maintained for 24 h. The cells were treated with the CPP-conjugated hFGF2 at a concentration of 0.1 nM or 1 nM and were incubated for either 30 min or 1 h. Thereafter, the cells were washed three times with fresh DMEM containing 1% FBS, and the medium was replaced with the normal medium. After five days of incubation, the effects of short-term treatment of CPP-conjugated hFGF2 on HDF were evaluated using the WST-1 cell viability assay.

To determine whether these effects are mediated by endocytosis and endocytosis recycling of cells, HDF was cultured and pretreated with 50 μM DYN for 24 h and short-term treatment of CPP-conjugated hFGF2 were performed as described above. In addition, to confirm whether this effect was caused by interactions between CPPs and cell membranes, the cells were washed three times with PBS containing 5 U/mL heparin after short-term treatment and replaced with normal medium. After 5 days, the results were analyzed by WST-1 cell viability assay.

### 4.11. Immunofluorescence Staining

For immunofluorescence staining, HDF was cultured on gelatin-coated coverslips to an appropriate density. The cells were fixed with 4% paraformaldehyde for 15 min and then permeabilized with 0.2% Triton X-100. After blocking in 3% bovine serum albumin (BSA, Bovogen, East Keilor, Australia) for 1 h, the coverslips were incubated for 1 h with primary antibodies diluted in 3% BSA. The coverslips were then washed with PBS containing 0.1% Tween 20 and were incubated for 30 min with Alexa Fluor 488-conjugated secondary antibodies and Alexa Fluor 647-conjugated phalloidin. The coverslips were then washed thoroughly and mounted on slides using a mounting solution with DAPI.

In order to assess the cell permeability of CPP-conjugated hFGF2, HDF was cultured and treated for 1 h with 1 nM CPP-conjugated hFGF2. After washing with PBS, the cells were detached with trypsin/EDTA and washed again with PBS. The cells were fixed with 4% paraformaldehyde for 15 min and then permeabilized with 0.2% Triton X-100. After blocking in 3% BSA for 1 h, the cells were incubated for 18 h at 4 °C with primary antibodies diluted in 3% BSA. After washing with PBS containing 0.1% Tween 20, the cells were incubated for 30 min with Alexa Fluor 488-conjugated secondary antibodies and Alexa Fluor 647-conjugated phalloidin. The cells were then thoroughly washed and mounted on slides using a mounting solution containing DAPI. All slides were analyzed by confocal microscopy. Fluorescence intensity was analyzed by ZEN 3.1 (blue edition) provided by Zeiss.

### 4.12. Western Blot Analysis

After 1 h of CPP-conjugated hFGF2 treatment at a concentration of 100 nM, HDF was washed by DMEM containing 1% FBS or PBS containing heparin. The cells were harvested according to the conditions for measuring intracellular CPP-conjugated hFGF2 over time. In order to obtain the cellular lysate, the cells were lysed on ice for 5 min in Cell Culture Lysis 1X Reagent (Promega, Fitchburg, WI, USA), which contained a mixture of protease and phosphatase inhibitors. Centrifugation at 15,000× *g* for 15 min at 4 °C removed any insoluble debris. A total of 20 μg of proteins in the isolated supernatants was separated using 12% SDS-PAGE and transferred to polyvinylidene difluoride membranes. The membranes were incubated for 1 h in 0.1% Tween 20 Tris-buffered saline (TBST) containing 5% skimmed milk at room temperature. The membranes were then incubated overnight at 4 °C in 5% skimmed milk-TBST with primary antibodies. After washing three times with TBST, the membranes were incubated for 1 h at room temperature in 5% skimmed milk-TBST with horseradish-peroxidase-conjugated secondary antibodies. Protein signals on the membranes were developed using ECL Western Blotting Substrate (Daeil Lab Service Co., Ltd., Seoul, Republic of Korea) and analyzed. All experiments were repeated three times.

To confirm the stability of CPP-conjugated hFGF2 over time in the experimental environment, HDF was resuspended in DMEM containing 10% FBS at a concentration of 1.0 × 10^5^ cells/mL and sonicated to make the cell lysate. CPP-conjugated hFGF2 was treated in normal medium or the medium containing the cell lysate at a concentration of 100 nM and samples were obtained over time. These samples were analyzed using his-tag antibody as described above.

### 4.13. ELISA

In order to measure the re-release of CPP-conjugated hFGF2, HDF was cultured and treated with 100 nM CPP-conjugated hFGF2 for 1 h before being washed twice with DMEM containing 1% FBS or PBS containing heparin. The cells were washed once again with DMEM containing 10% FBS. The amount of hFGF2 in the medium used for the final wash was defined as the initial (0 h) amount of hFGF2. Thereafter, the medium was obtained over time. Quantification of hFGF2 by the ELISA kit (BioLegend, San Diego, CA, USA) was performed in accordance with the manufacturer’s instructions.

### 4.14. Analysis of Re-Released hFGF2 Activity

HDF was treated for 1 h with CPP-conjugated hFGF2 at a concentration of 1 nM. After washing as described above, a conditioned medium was obtained over time and used for culturing newly seeded HDF. After five days of culturing, the effect of the re-released hFGF2 was confirmed by the WST-1 cell viability assay.

### 4.15. Statistical Analysis

All analyses were performed using SPSS Statistics software (version 25.0) (IBM Corp., Armonk, NY, USA) and GraphPad Prism 5 software (GraphPad Software Inc., La Jolla, CA, USA). Data are presented as the mean ± standard deviation (SD). Statistical analysis was conducted using analysis of variance and significance was defined as * p<0.05, ** p<0.01, and *** p<0.001 or **#** p<0, *##* p<0.01, and ### p<0.001.

## Figures and Tables

**Table 1:** Calculated physicochemical properties of Tat and Ara27.

Name	Amphipathicity	Length (aa)	MW (g/mol)	pI	Net Charge	ΔG Octanol (kcal/mol)	ΔG Interfacial (kcal/mol)
Tat	Nonamphipathic	10	1369	12.72	9	21.67	4.76
Ara27	Amphipathic	27	3334	11.56	8.9	26.05	4.83

**Table 2:** Sequence information of each component of the fusion proteins.

	Amino Acids Sequence
Tat	GRKKRRQRRR
Ara27	RNQRKTVRCFRCRQAGHWISDCRLKSK
hFGF2 *	AAGSITTLPALPEDGGSGAFPPGHFKDPKRLYCKNGGFFLRIHPDGRVDGVREKSDPHIKLQLQAEERGVVSIKGVCANRYLAMKEDGRLLASKCVTDECFFFERLESNNYNTYRSRKYTSWYVALKRTGQYKLGSKTGPGQKAILFLPMSAKS

* hFGF2, human fibroblast growth factor 2.

## Abbreviations

Arg_9_	Nona-arginine
BSA	Bovine serum albumin
cFGF2	Control fibroblast growth factor 2
CPPs	Cell-penetrating peptides
DAPI	4′,6-diamidino-2-phenylindole
DDS	Drug-delivery system
DMEM	Dulbecco’s modified Eagle’s medium
DYN	Dynasore
*E. coli*	*Escherichia coli*
EDTA	Ethylenediaminetetraacetic acid
ELISA	Enzyme-linked immunosorbent assay
FGFR	Fibroblast growth factor receptor
FITC	Fluorescein isothiocyanate
HDF	Human dermal fibroblast
hFGF2	Human fibroblast growth factor 2
LMWP	Low-molecular-weight protamine
MBP	Maltose binding protein
NOC	Nocodazole
ns	Not significant
NR-FGF2	Ara27-conjugated fibroblast growth factor 2
PBS	Phosphate-buffered saline
SD	Standard deviation
SDS-PAGE	Sodium dodecyl sulfate-polyacrylamide gel electrophoresis
TBST	Tween 20 Tris-buffered saline
tFGF2	Tat-conjugated fibroblast growth factor 2
